# *Ancylostoma ceylanicum* Hookworm in the Solomon Islands

**DOI:** 10.3201/eid2302.160822

**Published:** 2017-02

**Authors:** Richard S. Bradbury, Sze Fui Hii, Humpress Harrington, Richard Speare, Rebecca Traub

**Affiliations:** Central Queensland University, Rockhampton, Queensland, Australia (R.S. Bradbury);; University of Melbourne, Parkville, Victoria, Australia (S.F. Hii, R. Traub);; Pacific Adventist University, Atoifi, Malaita, Solomon Islands (H. Harrington);; Tropical Health Solutions, Townsville, Queensland, Australia (R. Speare);; James Cook University, Townsville (R. Speare)

**Keywords:** Ancylostoma ceylanicum, Solomon Islands, hookworm, cytochrome oxidase gene 1, cox-1 gene, multiplex PCR, helminth, parasites

## Abstract

Although hookworm is highly prevalent in the Solomon Islands, the species involved are unknown. We initiated this study in response to finding *Ancylostoma ceylanicum* hookworm in a peacekeeper in Australia who had returned from the Solomon Islands. Kato-Katz fecal surveys performed in 2013 and 2014 in 2 village groups in East Malaita, Solomon Islands, identified hookworm-positive samples. These specimens were tested by cytochrome oxidase 1 (*cox*-1) gene multiplex PCR and sequenced. Of 66 positive specimens, 54 (81.8%) contained only *Necator americanus*, 11 (16.7%) contained only *A. ceylanicum*, and 1 (1.5%) contained both species. *A. duodenale* was not found. Haplotype analysis of *cox*-1 sequences placed all human isolates (99% bootstrap support) of *A. ceylanicum* within the zoonotic clade rather than the human-specific clade. This study confirms that *A. ceylanicum* is endemic in the East Malaita region of this Pacific Island nation. The strain of the *A. ceylanicum* in this region can be shared among humans, dogs, and cats.

Hookworm disease, caused by blood-feeding worms of the small intestine, affects nearly 1 billion persons worldwide, with more than half of infections in the Asia-Pacific region (*1*). This disease causes iron deficiency anemia and malnutrition, leading to illness in pregnant women and children. In pregnant women, hookworm disease is associated with increased risks for death and poor neonatal outcomes, including low birthweight and increased deaths in infants. In children, substantial impacts on physical and intellectual development occur (*2**,**3*). Currently, *Necator americanus* and *Ancylostoma duodenale* hookworms are believed to be the main causative agents of hookworm disease globally.

Recently, *A. ceylanicum* hookworm infection was described in a soldier from Australia who had returned from the Regional Assistance Mission to the Solomon Islands, a peacekeeping mission (*4*). Until the past decade, human infection with *A. ceylanicum* was considered to be a rare zoonotic disease. However, several recent studies have found this species to be far more prevalent than previously reported, and it is now recognized as the second most common hookworm infection of humans in parts of Asia (*5*). The prevalence of this species in the Pacific Islands, and specifically in Melanesia, remains unexplored.

Isolated case reports of *A. ceylanicum* infection from the Pacific Islands were made in the early to mid-20th century. In 1929, *A. ceylanicum* infection was diagnosed in a 5-year-old child from Europe returning from long-term residence in the Shortland Islands (an island group in the north of the Solomon Islands) (*6*). Infection with *A. brazilense* (at that time synonymous with *A. ceylanicum*) was also described in 2 soldiers from Australia returning from service in Papua New Guinea during World War II (*7*) and in 9 servicemen from the Netherlands who had returned from West New Guinea in the early 1960s (*8*). *A. braziliense* and *A. ceylanicum* have similar morphologic features, with minor distinguishing points, but are distinct taxa (*9*). To our knowledge, *A. ceylanicum* infection has not been reported in the other Melanesian islands of Vanuatu or New Caledonia. Until the report of Speare et al. (*4*), no further human cases of *A. ceylanicum* infection were reported from the Solomon Islands or from any other Pacific Islands. In Australia, 2 autochthonous human infections of *A. ceylanicum* infection have been described (*10*).

In response to the case report of Speare et al. (*4*), we determined the species of hookworms, and, specifically, the prevalence of human *A. ceylanicum* infections, in the Solomon Islands, a group of islands southeast of Papua New Guinea and northeast of Australia, located within the Melanesian archipelago. Five soil-transmitted helminth (STH) surveys in this nation have been published (*11**–**15*). This survey aimed to determine the species of hookworm infecting communities in the remote East Malaita region of the province of Malaita.

## Methods

### Study Site

East Malaita is on the east coast of the island of Malaita in the Solomon Islands. The region has a wet equatorial tropical environment, with high rainfall. The average temperature is 27°C, and the weather is humid and hot at all times of the year. Two STH surveys, one in December 2013 and one in August 2014, were conducted in 2 villages of the province. Village 1 (Na’au) is on the mainland of Malaita, 0.95 km southeast of the major East Kwaio village of Atoifi (coordinates 8°52.729ʹS, 161°0.772ʹE). The village has a population of 195 persons and an average age of 30.1 years (median 28 years); 48.0% of the total population is female. Village group 2 (Kwai and Ngongosila) is found in adjacent coral atolls off the coast of East Malaita, 12.3 km north of Atoifi. Ngongosila is 0.83 km off the coast and Kwai is 0.55 km east of Ngongosila. At low tide, a land bridge is formed between the 2 islands. Kwai Island (8°46.20ʹS, 168°56.50ʹE) has a population of 458 persons, and Ngongosila (8°46.37ʹS, 168°56.28ʹE) has a population of 357 persons. The average age in village group 2 is 23.7 years (median 18 years), and 49.6% of the total population is female. All persons living in these villages are ethnic Malaitan Solomon Islanders. Although a small number of children and adults leave the villages to go to high school in larger population centers, most remain in their home village, to the extent that there is no underrepresentation of specific age groups in the village.

### Participant Recruitment

Initial discussions with village leaders and key members of the community were begun up to 12 months before the studies were performed. In the months prior to each study, discussions were held with the community. Community wide gatherings for leading to participant recruitment were held on December 14, 2013 for village 1 and on August 8, 2014 for village group 2. All residents were invited to participate. A census of residents was conducted before the survey to determine the number of persons and families on each island. Each person was assigned a code to preserve anonymity, and written informed consent was received from each participant or a parent or guardian. Each consenting person was provided with collection containers marked only with the participant code. Containers of fecal samples were left by the participants at the communal toilets in each village and were collected twice a day by the researchers.

### Hookworm Specimen Collection and Preservation

The presence of infecting STH was determined on the day of specimen collection by using a single Kato-Katz analysis (*16*). Kato-Katz slides were read within 1 hour and 30 minutes and at 4 hours after preparation. Samples positive for hookworms on Kato-Katz analysis were stored for later PCR analysis to determine the species of hookworm involved. Single fecal samples from egg-positive persons were preserved in 100% ethanol in a ratio of 1:1 and stored at room temperature until DNA extraction could be performed.

### Molecular Methods

Ethanol-preserved samples were rehydrated by being centrifuged at 500 × *g*; the ethanol supernatant was removed and replaced with sterile distilled, deionized water and incubated at 4°C overnight. DNA was then extracted with the Powersoil Kit (Mo Bio, Carlsbad, CA, USA), according to the manufacturer’s instructions. Extracted DNA was stored at −80°C until PCR was performed.

A previously published multiplex conventional PCR targeting the internal transcribed spacer region (ITS) 1, 5.8S, and ITS2 region of *Necator americanus* and *Ancylostoma* spp. was performed (*17*). We submitted PCR products of 380 bp corresponding to *Ancylostoma* spp. to Macrogen, Inc. (Seoul, South Korea), for purification and bidirectional DNA sequencing. We subjected samples that were positive for *A. ceylanicum* to a second published conventional PCR, targeting a 377-bp region of the *cox-*1 gene of *A. ceylanicum* for haplotype characterization (*18*).

We analyzed DNA sequences by using Finch TV version 1.4.0 (Geospiza, Inc., Billerica, MA, USA) and aligned them with BioEdit version 7.2.5 (http://www.mbio.ncsu.edu/bioedit/page2.html) with the *cox-*1 gene from *A. ceylanicum* Malaysia isolates (GenBank accession nos. KC247728/30/31/34/36/39/40/42–45, Pos Iskandar [Human], and Sg Bumbun [Human]); Cambodia isolates (GenBank accession nos. KF896595/97 and KF896600–KF896605);, and southern China isolates (GenBank accession nos. KP072071, KP072074, KP072080); *A. duodenale* (GenBank accession no. NC003415) and *A. caninum* (GenBank accession no. NC012309) isolates were also included in the analysis. We inferred the evolutionary history by the neighbor-joining method and computed evolutionary distances with the maximum composite likelihood method by base substitutions per site. The analysis involved 37 nt sequences. Codon positions included were 1st + 2nd + 3rd + noncoding. All positions containing gaps and missing data were eliminated. The final data set had 296 positions. We conducted evolutionary analyses in MEGA6 (http://www.megasoftware.net/).

### Statistical Analysis

We performed statistical analysis in Excel (Microsoft, Redmond, WA, USA) using the Student *t*-test (2-tailed) for comparison of the age of infected participants by the infecting species of hookworm. We used the χ^2^ test for gender but the Fisher exact test was used for source village and correlation to co-infection with other STH because the numbers were insufficient for a valid χ^2^ analysis. We considered any p value <0.05 to be significant.

### Ethical Approval

Ethical approval for this study was granted by the Atoifi Adventist Hospital Ethics Committee (approval no. AAH008). Reciprocal ethical approval was granted by the Central Queensland University Human Research Ethics Committee (approval no. H15/05–099).

## Results

A total of 65 fecal samples (33% participation rate) were received from village 1 and 576 fecal samples (71% participation rate) were received from village group 2. The prevalence of hookworms in village 1 and village group 2, according to the results of the Kato-Katz analysis, was 47.7% (31/65) and 24.1% (139/576), respectively.

The species of hookworm in 66 hookworm-positive samples was determined by multiplex ITS1, 5.8S, and ITS2 region PCR. Most samples, 81.8% (54/66), contained only *N. americanus* hookworm; 16.7% (11/66) contained only *A. ceylanicum* hookworm, and 1 sample (1.5%) contained a mixed infection with both *N. americanus* and *A. ceylanicum* hookworms. The species of hookworm infecting these persons had no major effect on the likelihood of infection with other STH; furthermore, we noted no association of age, sex, or village to a specific hookworm species (Table).

**Table:** Prevalence of *Ancylostoma ceylanicum* and *Necator americanus* among hookworm egg–positive fecal samples collected in East Kwaio, Solomon Islands, 2013–2014.

All patient samples	*A. ceylanicum*†	*N. americanus*†	Total	p value
Total no. samples	12	55	66	
Patient sex				
M	6	27	33	0.78
F	5	27	32	
ND	1	1	2	
Patient age, y				
Mean	30‡	32§	32	0.78
Median	30	34	32	
Range	2–65	5–80	2–80	
Village				
Village 1	4	12	16	0.46
Village group 2	8	42	50	
Other	0	1	1	
STH co-infection				
* Ascaris lumbricoides*	5	20	25	0.75
* Trichuris trichiura*	1	5	6	1.00
Both	0	5	5	0.58

*ND, no data; STH, soil-transmitted helminth. †Includes 1 mixed sample. ‡Two samples excluded from analysis because no data for age were recorded. §Four samples excluded from analysis because no data for age were recorded.

Of the 12 *A. ceylanicum* hookworm–positive samples, we successfully amplified the *cox-1* gene for 10. Haplotype analysis of *cox*-*1* sequences placed all human isolates (99% bootstrap support) within the clade comprising a mix of *A. ceylanicum* isolates sourced from humans, dogs, and cats in Malaysia, China, and Cambodia, as opposed to the clade comprising human-only isolates of *A. ceylanicum* (Figure).

**Figure:**
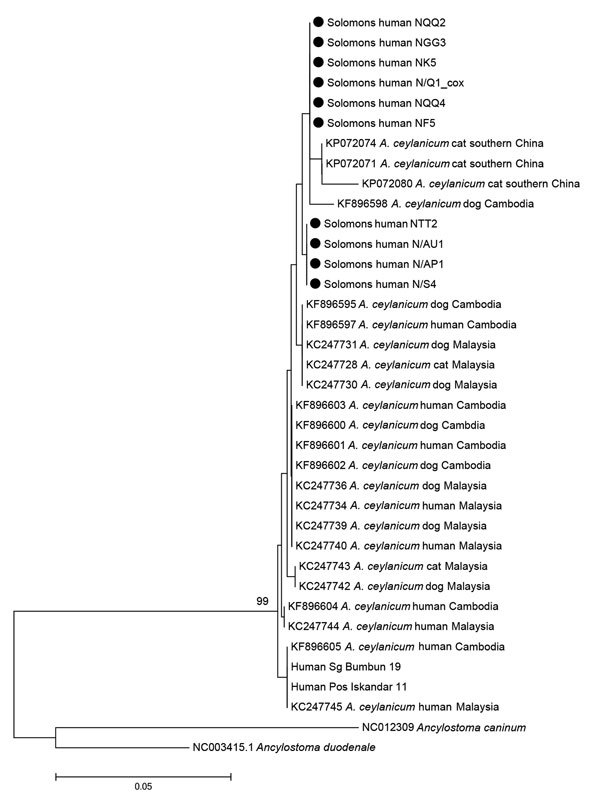
Phylogenetic tree obtained from neighbor-joining analysis of cytochrome oxidase 1 gene amplicons (296 bp) of *Ancylostoma ceylanicum* hookworms sourced from 10 humans in the eastern Solomon Islands (black circles) compared with reference isolates from Malaysia, China, and Cambodia, sourced from GenBank (accession numbers shown). Scale bar indicates nucleotide substitutions per site.

## Discussion

Human infection with *A. ceylanicum* hookworm is highly prevalent in the East Malaita region of the Solomon Islands, comprising 18.2% of tested samples found by the Kato-Katz method to contain hookworm eggs. Overall hookworm prevalence at Na’au was 47.7% and at Kwai-Ngongosila was 24.1%, and the prevalence of *A. ceylanicum* hookworm in tested samples at these villages was 11.9% and 3.9%, respectively. It appears that, as is the case in many Southeast Asia countries, *A. ceylanicum* is the second most common hookworm infecting humans in East Malaita. The presence of *A. ceylanicum* infection in the Eastern Malaita region of the Solomon Islands adds to the growing weight of evidence that *A. ceylanicum* is a widespread hookworm in humans and that this species may be present in other parts of Melanesia and the Pacific Islands. Indeed, the previously held assumption that human infection with this hookworm was a rare and unusual occurrence now appears false.

All *A. ceylanicum* isolates recovered in this study clustered within the zoonotic clade of this hookworm species, able to infect humans, dogs, and cats in China, Malaysia (*19*), and Cambodia (*18*). No isolates belonged to the second, more rare, clade also found in Southeast Asia, which, to date, consists only of human-infecting strains (*18**,**20*). Human settlement in the Pacific Islands is a more recent event than human colonization of Asia (*21*), and the human population density of the Pacific Islands is far lower than that in Southeast Asia. It is possible to propose a hypothesis that the human-adapted clade of *A. ceylanicum* might have evolved from the zoonotic clade in Asia, evolving to become specific to human hosts over an extended period of transmission only within that host species. Strains of *A. ceylanicum* brought to Melanesia with humans and their domesticated animals would be at a selective disadvantage to evolve from zoonotic to anthroponotic helminths because of the much lower human population density and relatively shorter time for evolution. Collection of multiple examples of each clade from throughout the Southeast Asian and Pacific regions, followed by analysis of evolutionary changes in mitochondrial DNA, might assist in clarifying this hypothesis. 

Dogs and cats are found in these rural villages. In a 2011 survey of Na’au, 17.5% of respondents reported owning or having >1 dogs in their household, and 40.8% reported similar close contact with cats (R. Speare, H. Harrington, unpub. data). In the East Malaita region of the Solomon Islands, this strain of *A. ceylanicum* may be both a zoonosis and an anthroponosis, transmitting in multiple directions among humans, dogs, and cats. Given the high prevalence in humans, it seems likely that if a wide selection of domestic cats and dogs were sampled, animal infections with this zoonotic clade of *A. ceylanicum* would also be discovered.

We did not recover *A. duodenale* hookworms from any of the 66 human infections sampled. This finding is consistent with the widely held assumption of early researchers that *A. duodenale* was a hookworm native to Europe, India, and China that had been introduced into the Pacific Islands by immigrants from those parts of the world. Early reports from American Samoa, Solomon Islands, Ellice Islands, Tonga, Cook Islands, and New Hebrides found exclusively *N. americanus* infection among the local populations, with *A. duodenale* hookworm seen only in immigrant populations from Europe, China, and India (*22**–**24*). Darling et al. (*24*) found infections with *A. ceylanicum*, but not *A. duodenale*, hookworm in native populations of Fiji in the 1920s but did not report the relative prevalence of these species. Walker and Bellmaine (*7*) reported a large number of *A. duodenale* infections in servicemen from Australia returning from Papua New Guinea during World War II, but this hookworm was common in northern Australia at that time (*25*) and may have also been imported from the Northern Hemisphere with European colonization or acquired from other soldiers from Australia during the New Guinea campaign.

Several isolated cases of *A. ceylanicum* infection from the Pacific Islands and, more specifically, Melanesia have been reported previously, in which adult worms were recovered and identified (*4**,**6**,**24*). Only 1 study in the Pacific region (specifically, in Australia) has used PCR for species identification; this study found a relatively high percentage of *A. ceylanicum* infection (29%), although the sample size of autochthonous hookworms was low (n = 7) (*10*). 

Given the established presence of human *A. ceylanicum* infections in Southeast Asia, Australia, and the Solomon Islands, and the rare historical reports of cases from Papua New Guinea and Fiji (*8**,**24*), it seems likely that this infection is common in other countries in the wider Pacific region, and particularly in Melanesia. Although numerous studies of hookworm prevalence in the regions have been performed previously, in very few were the species of hookworm identified. Those studies that did specific taxonomy almost exclusively used morphology of filariform larvae to differentiate *A. duodenale* from *N. americanus*. Although *N. americanus* hookworm lacks the prominent constriction of the intestine at the esophageal junction that is seen in filariform larva of *A. duodenale*, as well as several other subtle morphological differences, morphological differentiation of 2 species is established primarily by the far more prominent striations of the posterior sheath of *N. americanus*. These striations are not readily visible on the posterior sheath of *A. duodenale* larvae (*26*). The filariform larva of *A. ceylanicum* share the posterior sheath striations seen in *N. americanus* (*27*) and thus may easily have been misidentified as *N. americanus* in studies in which only the morphology of these larvae in culture was used for identification. Larger surveys using PCR for species identification to further elucidate the prevalence and extent of *A. ceylanicum* infections throughout the Pacific are warranted.

This study did not record data on clinical data such as hemoglobin, eosinophilia, and nutritional status among participants for comparison of the relative impact of infecting hookworm species on these indices. Experimental infection of 2 human volunteers with *A. ceylanicum* hookworm by Carrol and Grove (*28*) found that eosinophilia occurred initially, declined after 4 weeks, and then completely resolved despite ongoing infection. These volunteers also showed no abnormality in hemoglobin or other blood count parameters. This pattern is similar to that seen with experimental infections with *N. americanus* hookworm (*29*). In contrast, Anten and Zuidema (*8*) noted marked eosinophilia and iron deficiency anemia among Dutch servicemen returning from New Guinea who were infected with *A. ceylanicum* hookworm. Given the high prevalence of *A. ceylanicum* infection in many parts of the world, studies of the clinical and nutritional effects of this soil-transmitted helminth are needed to determine the role and potential pathogenicity of infection.

This study was not specifically intended to act as a prevalence survey; rather, it was an attempt to define whether *A. ceylanicum* might be present in Melanesia. However, the percentage of hookworm-positive samples recovered is likely to be broadly representative of the total hookworm burden in those villages. Hookworm can cause mild diarrhea in symptomatic patients, which may introduce a slight bias in prevalence data. However, PCR is more sensitive than single-sample microscopy for the detection of hookworm (*18*). Had this method been performed on all fecal samples, it is almost certain that a higher number of positive samples would have been found. Furthermore, the relative frequency of each hookworm species in the random subset of microscopy-positive samples preserved and tested by PCR in Australia may not reflect the true prevalence in the source population. Although a degree of correlation of species prevalence results might be reasonably expected, it is not known whether *A. ceylanicum* hookworms produce relatively fewer or more eggs per day than *N. americanus* hookworms or if one species causes more severe diarrhea than the other. Each of these factors could have led to a degree of bias in the selection of microscopy-positive samples for later species analysis.

In summary, this study presents findings showing that *A. ceylanicum* may be the second most common human hookworm infection in the East Malaita region of the Solomon Islands, after *N. americanus*. By contrast, infection with *A. duodenale* hookworm appears to be absent from this region. We recommend that further work be done to investigate the prevalence of this species in humans and their domestic animals throughout the Pacific Island nations and territories and that its clinical significance as a human pathogen be elucidated as soon as feasible. This finding also highlights that hookworm control programs in Solomon Islands would benefit from considering a One Health approach, because, to be successful, these programs may have to control hookworms in humans, dogs, and cats simultaneously (*5*).
